# Systemic Lupus Erythematosus Presenting as Acute Lupus Pneumonitis during Pregnancy

**DOI:** 10.1155/2020/8839410

**Published:** 2020-12-21

**Authors:** Marlene Marte Furment, Suyansh Sharma, Sangeetha Pabolu

**Affiliations:** ^1^Internal Medicine Department, Saint Peter's University Hospital, Rutgers Robert Wood Johnson Medical School Program, New Brunswick, NJ, USA; ^2^Rheumatology Department, Saint Peter's University Hospital, Rutgers Robert Wood Johnson Medical School Program, New Brunswick, NJ, USA

## Abstract

**Introduction:**

This is a case of new-onset systemic lupus erythematosus (SLE) manifesting as acute pneumonitis during pregnancy. No prior reports have documented pneumonitis as the presenting manifestation of SLE in pregnant women. *Case Presentation*. A 23-year-old pregnant female presented with high-grade fever, cough, arthralgias, and respiratory failure. Infectious workup was negative. She was positive for ANA, anti-dsDNA, anti-SSA, hypocomplementemia, and pulmonary infiltrates, supporting the diagnosis of SLE and pneumonitis. The patient received methylprednisolone achieving adequate clinical and serological response.

**Conclusion:**

When SLE patients present with fever, cough, and respiratory failure, pulmonary infiltrates should raise the suspicion of pneumonitis in the absence of infection and hemorrhage. Even though acute lupus pneumonitis (ALP) is rare and seen only in 2% of SLE patients, a high index of suspicion aids in prompt diagnosis of this life-threatening condition. Also, positive anti-SSA antibodies may be associated with lupus pneumonitis.

## 1. Introduction

We report a rare case of a young female diagnosed with new-onset SLE during her pregnancy, manifested as acute lupus pneumonitis (ALP). ALP is a life-threatening and uncommon manifestation of SLE that requires prompt diagnosis and treatment. There are no case reports in the literature documenting pneumonitis as the presenting manifestation of SLE in pregnant population.

## 2. Case Presentation

A 23-year-old Hispanic female at 19 weeks of gestation, with medical history significant for preeclampsia and mild intermittent asthma, presented to the Emergency Room complaining of fever, dry cough, and shortness of breath for a week. The patient also reported painful swelling in her hands and feet associated with morning stiffness. She had tried acetaminophen at home which did not relieve her symptoms. Initial examination was remarkable for tachycardia (103 beats/min), tachypnea (20 breaths/min), and fever (101.8°F). Her lung examination was clear with equal air entry bilaterally. Joint exam showed swelling, tenderness, and warmth of bilateral ankles, knees, proximal interphalangeal joints, and metacarpal phalangeal joints. Laboratory studies were significant for lymphopenia (white blood cell count of 2.6 × 10/µL) and anemia (hemoglobin of 10.5 g/dL). Chest X-ray on admission did not show abnormalities.

Bilateral lower-extremity venous duplex studies did not show evidence of deep vein thromboses. Additionally, left knee arthrocentesis ruled out septic arthritis.

She was started empirically on ceftriaxone and azithromycin for possible community-acquired pneumonia. Her symptoms did not improve. C-reactive protein (CRP) and erythrocyte sedimentation rate were elevated (13 mg/L and 59 mm/h, respectively). Low complement levels were noted (C3 of 36 mg/dL and C4 of <8 mg/dL). The ANA test was positive. On day 5 of admission, she became tachypneic and hypoxic, saturating 84% on room air despite noninvasive ventilation. She was upgraded to the Intensive Care Unit where she required a high-flow nasal cannula.

**Figure 1:**
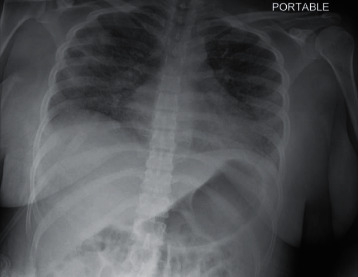
Portable chest X-ray film from our patient on day 5 of admission showing bilateral multifocal airspace opacities consistent with pneumonitis.

Repeat chest X-ray showed new multifocal bilateral airspace opacities but no pleural effusions or pneumothorax (Figure 1).

Extensive infectious workup for viral, bacterial, and parasitic causes was negative. Notably, sputum cultures, blood cultures, and cultures for SARS-CoV-2, HIV, CMV, EBV, parvovirus B-19, hepatitis, and West Nile virus were negative. Legionella and Streptococcus urine antigens, Mycoplasma pneumoniae, QuantiFERON for tuberculosis, Lyme, Ehrlichia, Anaplasma, PCR for influenza A (subtypes H1 and H3), adenovirus, parainfluenza, and rhinovirus were also negative.

The patient was started empirically on IV methylprednisolone 60 mg daily for probable pneumonitis. This led to significant improvement of symptoms as her fever, tachypnea, hypoxia, cough, and arthralgias had resolved within 24 hours.

On further investigation, she was found to have positive anti-dsDNA (1 : 160 titers) and anti-SSA antibodies. CT chest was not pursued, given the risk of radiation exposure in pregnancy.

She never developed hemoptysis or significant drop in hemoglobin to favor of alveolar hemorrhage. The patient was diagnosed with new-onset SLE as per the EULAR/ACR 2019 criteria as she presented with fever, leukopenia, arthritis, pneumonitis, low complements, and positive serological lupus studies (ANA and anti-dsDNA). She was started on hydroxychloroquine 200 mg twice daily, and intravenous corticosteroids were switched to oral prednisone on the fourth day of IV methylprednisolone. This achieved adequate clinical and serological response as her CRP, hypocomplementemia, and pancytopenia significantly improved within 5 days.

## 3. Discussion and Conclusions

New-onset systemic lupus erythematosus during pregnancy usually presents with hematologic and renal manifestations [1]. Uniquely, our patient presented with acute lupus pneumonitis (ALP). ALP is a rare SLE complication that affects around 2% of these patients [2, 3]. It presents with fever, cough, dyspnea, and hypoxemia in a patient with suggestive symptoms of lupus such as arthralgias, fatigue, and malar rash. Data suggest a high mortality of up to 50% in lupus pneumonitis despite adequate treatment [4, 5].

Chest radiographs commonly reveal unilateral or bilateral patchy opacities mostly in lung bases and may be associated with pleural effusion or atelectasis [6]. Other pleuropulmonary manifestations in SLE include pleuritis (which is the most common), diffuse alveolar hemorrhage, interstitial lung disease, bronchiolitis obliterans, pulmonary hypertension, pulmonary embolism, and shrinking lung syndrome [7]. Pleuropulmonary manifestations in SLE were first described by Sir William Osler in 1904 upon noticing persistent infiltrates in lupus diathesis that were unrelated to infection [2].

The underlying pathophysiology of ALP is not clear yet. Matthay et al. [2] identified through pathology reports the presence of acute alveolar and hyaline membrane damage with interstitial edema, alveolitis, and arteriolar thrombosis. Like our case, some studies have suggested that serum anti-SSA antibodies are likely associated with ALP [5, 8, 9]. However, their causative role in ALP is not clear, with other studies showing controversial results [10, 11].

The cornerstone in the management of lupus pneumonitis is systemic corticosteroids. Although steroids in pregnancy increase the risk for premature rupture of membranes, diabetes, and preeclampsia, they remain the first-line therapy for ALP [12]. An acceptable approach is the use of oral prednisone 1–1.5 mg/kg/d in divided doses. Intravenous corticosteroids (1 g methylprednisolone per day for 3 days) may also be used if no clinical response is noticed within 72 hours [4]. Hydroxychloroquine use during pregnancy has been proven to decrease SLE disease activity and relapses; however, its use for ALP has not been established. It also decreases the risk of congenital heart block and neonatal lupus in those at risk with positive anti-SSA antibodies, like our case [13, 14]. Azathioprine may also be used and has been generally considered safe during pregnancy although it has been associated with late developmental delays in offspring [15, 16].

In conclusion, new-onset SLE during pregnancy may manifest as ALP. Even though rare, ALP needs a high index of suspicion for prompt diagnosis and treatment due to its severity. There are no definitive guidelines established in the literature for the diagnosis of ALP. It should be suspected in patients with fever, cough, and respiratory failure not responding to antibiotics, with imaging findings of parenchymal pulmonary involvement and positive serologies for lupus. As in our case, positive anti-SSA antibody may be associated with ALP and needs further studies to confirm. Management has not been studied in randomized controlled trials, but it is generally accepted to treat with systemic corticosteroids along with immunosuppressive therapy.

## Abbreviations

ACR: American College of Rheumatology
ALP: Acute lupus pneumonitis
ANA: Antinuclear antibody
Anti-dsDNA: Anti-double-stranded DNA
Anti-SSA: Anti-Sjögren's syndrome-related antigen A
CMV: Cytomegalovirus
CRP: C-reactive protein
EBV: Epstein–Barr virus
EULAR: European League Against Rheumatism
HIV: Human immunodeficiency virus
PCR: Polymerase chain reaction
SARS-CoV-2: Severe acute respiratory syndrome coronavirus 2
SLE: Systemic lupus erythematosus.
